# Mastocytome solitaire de l'enfant: à propos d'un cas

**DOI:** 10.11604/pamj.2019.32.45.14356

**Published:** 2019-01-28

**Authors:** Fatima-Zahra Agharbi

**Affiliations:** 1 Hôpital Civil Tétouan, Tétouan, Maroc

**Keywords:** Mastocytoma, solitary, mastocytosis, children, Mastocytoma, isolated, mastocytosis, children

## Abstract

Le mastocytome solitaire est la forme la plus fréquente de mastocytose chez l'enfant. Son pronostic est bon comme toutes les autres formes de mastocytoses de l'enfant avec possibilité de régression spontanée. Les dermocorticoïdes peuvent accélérer cette régression comme c'est le cas chez notre patient.

## Abstract (en)

Isolated mastocytoma is the most common form of mastocytosis in children. Prognosis is good, as in all other forms of mastocytosis in children, with possibility of spontaneous regression. Dermocorticoids can accelerate this regression, as it is the case for our patient.

## Introduction

Les mastocytoses sont des maladies rares liées à l'accumulation et à la prolifération anormale des mastocytes dans un ou plusieurs organes. La peau est l'organe le plus fréquemment touché [1]. Contrairement à l'adulte cette affection est le plus souvent indolente chez l'enfant avec une atteinte cutanée isolée. Le mastocytome solitaire exceptionnel chez l'adulte est la forme la plus fréquente chez l'enfant dont nous rapportons un cas chez un nourrisson de 4 mois [2].

## Patient et observation

**Figure 1:**
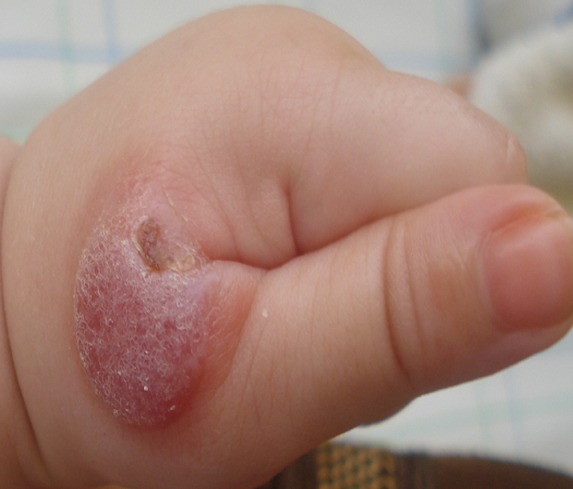
Nodule jaune orangé du dos de la main

**Figure 2:**
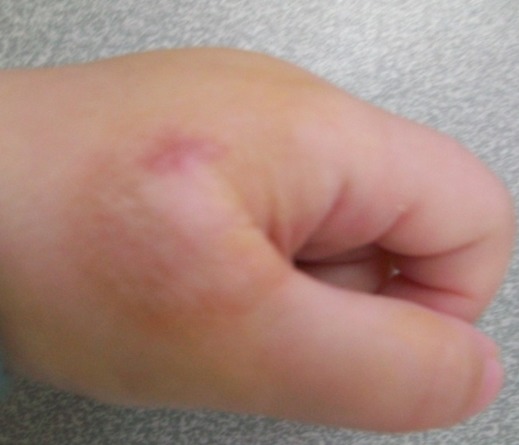
Affaissement du nodule après traitement par dermocorticoïdes

Il s'agit d'un nourrisson âgé de 4 mois, sans antécédent pathologiques notables, qui présentait depuis l'âge de 2 mois, une lésion érythémateuse du dos de la main droite prurigineuse avec des épisodes de flushs du tronc et du visage rapportés par la famille au cours desquels la lésion devient turgescente parfois bulleuse. L'examen dermatologique trouvait un nodule de couleur jaune orangée, consistance ferme, mesurant 3 cm sur 2 cm, siégeant au niveau du dos de la main droite (Figure 1). Le signe de Darier était positif. Le reste de l'examen somatique était sans particularités. Le diagnostic de mastocytome solitaire a été évoqué et confirmer par l'histologie. Le patient a été mis sous dermocorticoïdes classe forte sous occlusion avec bonne évolution (Figure 2).

## Discussion

Le mastocytome solitaire est une forme de mastocytose papulo-nodulaire. C'est la forme la plus fréquente de mastocytose chez l'enfant chez qui elle survient avant l'âge de 2 ans, le plus souvent dans les 3 premiers mois. Elle se présente sous forme de nodule volontiers unique (d'où l'appellation mastocytome solitaire), hémisphérique, ferme, de 1 à 4 cm de diamètre, bien limité, de couleur jaunâtre rosée à brune, de siège ubiquitaire. Des poussées inflammatoires urticariennes voire vésiculo bulleuses peuvent survenir spontanément ou bien après effort physique, stress émotionnel, prise alimentaire ou médicamenteuse. Le frottement de la lésion peut également déclencher ces poussées inflammatoires représentant ainsi le signe de Darier qui est inconstant mais pathognomonique des mastocytoses. Des épisodes de flushs avec prurit peuvent également être observés avec les mêmes facteurs déclenchant la dégranulation mastocytaire [2-5].

## Conclusion

Le mastocytome solitaire est la forme de mastocytose cutanée la plus fréquente chez l'enfant dont le pronostic est favorable avec possibilité de régression spontanée. Les dermocorticoïdes peuvent accélérer cette régression comme c'est le cas chez notre patient [6].
